# Scaling of cotyledon and primary leaf mass versus area in *Acer platanoides* seedlings under different light conditions

**DOI:** 10.1093/aobpla/plae054

**Published:** 2024-09-25

**Authors:** Jinfeng Wang, Bader O Almutairi, Lin Wang, Peijian Shi, Weihao Yao, Ülo Niinemets

**Affiliations:** Co-Innovation Centre for Sustainable Forestry in Southern China, Bamboo Research Institute, Nanjing Forestry University, 159 Longpan Road, Nanjing 210037, China; College of Science, King Saud University, P.O. Box 2455, Riyadh 11451, Saudi Arabia; College of Life Sciences, Sichuan University, 29 Wangjiang Road, Chengdu 610065, China; Co-Innovation Centre for Sustainable Forestry in Southern China, Bamboo Research Institute, Nanjing Forestry University, 159 Longpan Road, Nanjing 210037, China; Co-Innovation Centre for Sustainable Forestry in Southern China, Bamboo Research Institute, Nanjing Forestry University, 159 Longpan Road, Nanjing 210037, China; College of Science, King Saud University, P.O. Box 2455, Riyadh 11451, Saudi Arabia; Institute of Agricultural and Environmental Sciences, Estonian University of Life Sciences, Kreutzwaldi 1, 51006 Tartu, Estonia

**Keywords:** Cotyledon, diminishing returns, light level, scaling theory, seedling

## Abstract

Cotyledons play an important role in early seedling establishment. However, relative to primary leaves, cotyledons tend to have a different investment-on-return strategy. To detect the potential differences in the mass (*M*) versus area (*A*) scaling relationships between cotyledons and primary leaves in different light environments, a total of 75 *Acer platanoides* seedlings were sampled at an open site (*n* = 52; light availability: 74 ± 5 %) and a shaded site (*n* = 23; light availability: 4.2 ± 1.2 %). Reduced major axis regression protocols were used to fit the *M* versus *A* scaling relationships of primary leaves and cotyledons. The bootstrap percentile method was used to test the significance of the differences in the scaling exponents of *M* versus *A* between the two light environments. The scaling exponents of cotyledons at both two sites, as well as the primary leaves at the shade site, were greater than unity indicating ‘diminishing returns’, while the scaling exponent of primary leaves at the open site was smaller than unity indicating ‘increasing returns’. The data collectively indicated light-dependent shifts in support investments and differences in the function of cotyledons and primary leaves. Average leaf structural traits displayed significant differences between the two light environments in accordance with the premium in enhancing photosynthetic capacity in high light and light interception in low light. Although the trait responses to light availability were similar for primary leaves and cotyledons, primary leaves were more responsive to light availability, indicating lower plasticity of cotyledons in response to light levels. These results advance our understanding of the roles of cotyledons and primary leaves in the life history of seedlings in different forest light environments.

## Teaser

Cotyledons and primary leaves show distinct growth strategies in *Acer platanoides* seedlings under different light conditions. A study of 75 seedlings revealed that in shaded environments, both cotyledons and primary leaves exhibited a ‘diminishing returns’ pattern in mass versus area scaling, while primary leaves at open sites showed ‘increasing returns’. Primary leaves were more responsive to light than cotyledons, highlighting their greater adaptability. These findings improve our understanding of how cotyledons and primary leaves contribute to seedling success in varying light environments.

## Introduction

Seedling stage is a highly sensitive developmental period in plant life, playing a critical role in plant survival and establishment (Harper 1977; Westoby *et al.* 2002; Rajjou *et al.* 2012; Spasojevic *et al*. 2014). Seedlings face asymmetric competition from larger individuals and must defend themselves against herbivores and pathogens in the forest (Kitajima and Myers 2008). Therefore, growth and allocation strategies at the seedling stage are particularly important. Cotyledons, as the first leaves post-germination are crucial for early plant growth, providing nutrients and carbon from storage tissues and carbon through photosynthesis (Webb and Gorham 1964; Lovell and Moor 1970; Penny *et al*. 1976; Kitajima 2003; Hanley and May 2006; Zhang *et al*. 2008; Zheng *et al*. 2011*a*). Cotyledon photosynthetic responses, along with those of primary leaves, strongly influence the later stages of seedling development and initial growth in higher plants (Penny *et al*. 1976). It has been demonstrated that the cotyledons have developed a variety of defense mechanisms to adapt to and resist different types of stresses over a long evolutionary period (Krüger *et al*. 2002; Yan *et al*. 2010; Luan *et al*. 2015; Ren *et al*. 2015). For instance, the accumulation of Na^+^ in cotyledons helps reduce salt stress damage to other parts of the seedling in salt-stressed *Suaeda physophora* (Zhou *et al.* 2014).

Scaling theory describes the relationships and changes between different traits, providing a theoretical foundation for patterns of biomass partitioning, and revealing how plants use resources and adapt to diverse environments (West *et al*. 1997; Shingleton 2010). Scaling occurs across and within organs, evident in various plant life-history traits (Primack 1987; Niklas and Enquist 2002; Cheng *et al*. 2014; Chen *et al*. 2020; Tan *et al.* 2024). For example, with increasing latitude, the mass ratios of pappus and seed coat in a whole seed of *Geropogon hybridus* decreased, whereas the mass ratio of seed reserve increased (Chen and Giladi 2018). Qiu *et al*. (2021) observed that some tree species grow faster in height when they are young, whereas they prioritize diameter at maturity. The site-specific and idiosyncratic variation in plant biomass allocation has long been recognized, but there has been limited research to further investigate whether this results in changes in allometric relationships (Callaway *et al*. 1994; Bazzaz and Grace 1997). Enquist and Niklas (2002) acknowledged that their predictions might be inapplicable to seedlings less than 1 year old, but once an individual plant becomes photosynthetically self-sufficient and has exhausted its maternal contribution to early seedlings development, the allometric relationships agreed with theoretical predictions.

Scaling theory has been applied to plant seedlings and saplings in several studies. Niklas (1995) found that the scaling exponent of tree height versus trunk diameter in 27 *Robinia pseudoacacia* trees of different age and height was > 1.0 for small and young trees and decreased to 2/3 and then to 1/2 as tree size and age increased. Chen and Li (2003) concluded, in their study of seedlings of biennial plants, that the scaling exponent of photosynthetic rate versus total biomass differed under different relative soil water contents within the same species.

The primary leaves and cotyledons of seedlings are essential for seedling development, but their scaling has received little attention. On average, increases in lamina area fail to keep pace with increases in lamina mass, i.e. increasing biomass investments do not result in proportional (one-to-one) increases in lamina area, a relationship called ‘diminishing returns’ (Niklas *et al*. 2007). This has important implications for biomass allocation patterns and vegetative growth as well as other aspects of life-history strategies. Therefore, the aim of this research was to test whether the law of diminishing returns is equally valid for cotyledons and primary leaves in seedlings of Norway maple (*Acer platanoides*, Sapindaceae), a species with conspicuous cotyledons in early seedling development.

Light is the key environmental resource for plants, providing energy for photosynthesis, and triggering multiple photomorphogenic responses (Niinemets 2007*a*; Li *et al*. 2020). Under different light environments, plants adjust their morphological structure by altering the biomass distribution among roots, stems and leaves as well as by modifying the anatomy of leaves to change the light-exposed area (Evans and Poorter 2001; Morris *et al*. 2017; Burko *et al*. 2022). Leaf display and light capture efficiency depend on canopy architecture, size of the leaves and biomass investment in leaf lamina and lamina support (Falster and Westoby 2003; Pearcy *et al*. 2004, 2005; Niinemets *et al*. 2007*b*). Leaf size, which can be quantified by lamina area and mass, is a particularly influential functional trait (Niinemets *et al*. 2007*b*). There is little information on how light affects the allocation of biomass in seedling primary leaves and cotyledons, and it is still unclear how light levels affect cotyledon and primary leaf traits. As cotyledon structure is determined primarily during seed development (Chandler 2008), while primary leaves develop in the actual light environment of seedling, it is likely that primary leaves respond to differences in light availability more plastically than cotyledons. In the current study, we studied seedlings grown at an open site (forest gap) and a shaded site (forest understory) to investigate whether varying light levels can lead to differences in leaf traits of the seedlings. Our main aim was to study biomass investments in the lamina of primary leaves and cotyledons in *A. platanoides*, a shade-tolerant European forest species that has been widely introduced to temperate and boreal ecosystems worldwide. This species dominates the regeneration layers and young tree classes in many urban woodlots and is a canopy species in natural European late-successional forest stands (Webb and Kaunzinger 1993). By examining seedlings from two populations, one from an open site and the other from a shaded site, we aimed to address the following questions: (i) Do the primary leaves and cotyledons of seedlings scale similarly and follow the law of ‘diminishing returns’? (ii) Is there a difference in the leaf mass versus area scaling exponent of cotyledons, and primary leaves of seedlings grown in different light environments? (iii) How do cotyledon and primary leaf structural traits differ? and (iv) Do the differences in cotyledon and primary leaf structural traits vary among plants grown in different light environments?

## Materials and Methods

### Study sites and plant collection

During 10–15 May 2023, naturally seeded current-year seedlings of *A*. *platanoides* were collected from two sites with different light availability in Tartu, Estonia. The open site was a residential garden with mixed evergreen conifer-deciduous broad-leaved woody vegetation (58°21ʹ33.84″ N, 26°46ʹ49.80″ E; elevation 36 m), and the shaded site was a mixed evergreen conifer-deciduous broad-leaved forest (58°21ʹ37.44″ N, 26°46ʹ51.60″ E; elevation 36 m). The tree composition of the two sites was similar. The key overstory species in the open site were naturally-seeded native species *Populus tremula*, *Betula pendula*, *Quercus robur* and *Pinus sylvestris*, and planted exotic species *Malus domestica* and *Picea pungens*, whereas the shaded site was dominated by native species *Prunus padus*, *P. tremula*, *B. pendula*, *Corylus avellana*, *A*. *platanoides*, *P. sylvestris* and *Picea abies*. From the ecological point of view, the open site is a moderately big forest gap (about 7 × 10 m), whereas the shaded site is a forest understory. The two sites are about 100–200 m apart, have a similar soil type (sod-podzolic soil) and the two sites have not received any fertilizer for at least the last 20 years. The seedling roots at the time of sampling (3–5 cm long) were primarily within the litter and turf layer (O layer), whereas the taproot tip of the seedlings started to reach the topsoil (A layer). At the time of sampling, the herbaceous layer was weakly developed and the surface was mainly covered by a moss layer.

Hemispheric photography was employed to characterize light availability at both sites. Five hemispheric photographs were taken above the seedlings using a Nikon Coolpix 990 camera (Nikon Corporation, Minato-Ku, Tokyo, Japan) equipped with FC-E8 fish-eye conversion lens. The Gap Light Analyzer software (https://www.caryinstitute.org/science/our-scientists/dr-charles-d-canham/gap-light-analyzer-gla) (Frazer *et al*. 1999) was used to estimate the percentage of diffuse solar radiation incident to the seedlings from the hemispheric images according to manufacturer’s instructions. The original RGB (Red-Green-Blue) image was converted to a black-and-white image using the blue channel. Light availability was estimated to be 74 ± 5 % (mean ± standard error) at the open site and 4.2 ± 1.2 % at the shaded site. Given that leaf structural and physiological traits respond asymptotically to light availability and saturate at light availabilities of 30–50 % (Poorter *et al.* 2009, 2010; Niinemets *et al.* 2015; Keenan and Niinemets 2017), we consider the light availability at the open site as representative to gain insight into the leaf acclimation capacity to high light.

At the time of sampling, the plants were ca. 20 days old with intact non-senescent cotyledons and with two primary leaves. A total of 52 plants were excavated from the open site, while 23 plants were collected from the shaded site. The plants were delicately removed from the turf, placed in plastic bags with wet filter paper, and promptly transported to the laboratory for measurements.

### Leaf mass measurement and image processing

**Figure 1.**
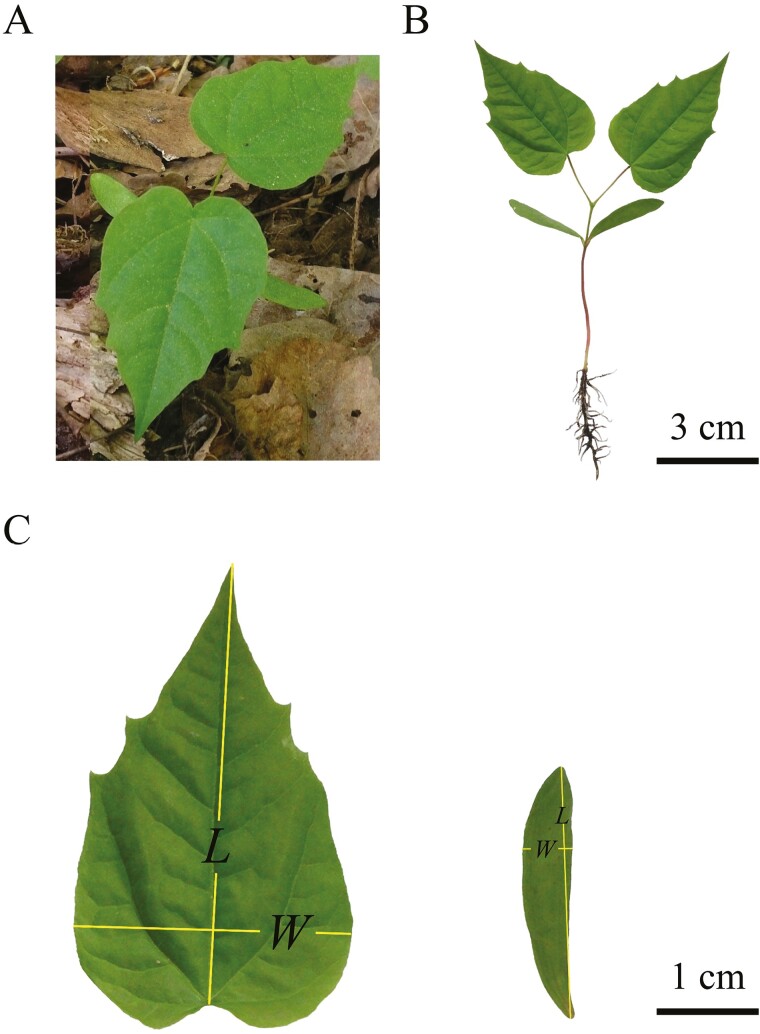
Representative photograph of an *Acer platanoides* seedling in its natural habitat (A), a scanned image of the whole seedling (B) and a representative primary leaf (left) and cotyledon (right) of an *A*. *platanoides* seedling (C). The measurements of length and width of primary leaf and cotyledon are shown in (C).

The pairs of cotyledons and primary leaves of *A*. *platanoides* are opposite and arranged in two planes; the cotyledon and primary leaf planes are almost horizontal and rotated about 90° relative to the plane tangents running through the tips of cotyledons and primary leaves (90° rotation relative to the stem; Fig. 1A). To facilitate scanning by a flatbed scanner, the upper portion of the plant with the primary leaves was rotated clockwise by 90°. The opposite cotyledons and primary leaves were then numbered before scanning. The entire seedling was scanned at 600-dpi resolution using a Canon i-SENSYS MF5490 multifunctional laser printer/scanner (Fig. 1B for a representative scanned image of an entire seedling). After scanning, the primary leaves and cotyledons were meticulously separated from the plant. Two opposite primary leaves and two opposite cotyledons were oven-dried to a constant mass at 70 °C (for at least 48 h), and the dry mass (*M*) of each primary leaf and cotyledon was determined to the nearest 0.1 mg using an electronic balance.

Adobe Photoshop CS2 (version 9.0; Adobe, San Jose, CA, USA) was used to obtain black and white images of primary leaf and cotyledon edges, which were saved as bitmap images at a 600-dpi resolution. The Matlab (version ≥ 2009a; MathWorks, Natick, MA, USA) procedure developed by Shi *et al*. (2018) and Su *et al*. (2019) was used to obtain the planar coordinates of primary leaf and cotyledon boundary points. The lamina measures, including lamina length (*L*), width (*W*) and area (*A*) of primary leaves and cotyledons, were calculated by ‘bilat’ function in the ‘biogeom’ package (version 1.4.1; Shi *et al*. 2022) in R (version 4.3.2; R Core Team 2023). The lamina length of the primary leaf was defined as the distance from the base to the tip of the lamina, this is approximately equal to the length of the lamina midrib (Fig. 1C, Sack *et al.* 2013*a*). The raw data are provided [see Supporting Information—Table S1], and the acronyms of all measured traits are defined in Table 1.

**Table 1.** Acronyms and abbreviations for the variables.

Abbreviation	Definition
*M*_OPL_	Lamina dry mass of primary leaves sampled at the open site
*M*_SPL_	Lamina dry mass of primary leaves sampled at the shaded site
*M*_OC_	Lamina dry mass of cotyledons sampled at the open site
*M*_SC_	Lamina dry mass of cotyledons sampled at the shaded site
*A*_OPL_	Lamina area of primary leaves sampled at the open site
*A*_SPL_	Lamina area of primary leaves sampled at the shaded site
*A*_OC_	Lamina area of cotyledons sampled at the open site
*A*_SC_	Lamina area of cotyledons sampled at the shaded site
*L*_OPL_	Lamina length of primary leaves sampled at the open site
*L*_SPL_	Lamina length of primary leaves sampled at the shaded site
*L*_OC_	Lamina length of cotyledons sampled at the open site
*L*_SC_	Lamina length of cotyledons sampled at the shaded site
*W*_OPL_	Lamina width of primary leaves sampled at the open site
*W*_SPL_	Lamina width of primary leaves sampled at the shaded site
*W*_OC_	Lamina width of cotyledons sampled at the open site
*W*_SC_	Lamina width of cotyledons sampled at the shaded site
LMA_OPL_	Dry mass per unit area of primary leaves sampled at the open site
LMA_SPL_	Dry mass per unit area of primary leaves sampled at the shaded site
LMA_OC_	Dry mass per unit area of cotyledons sampled at the open site
LMA_SC_	Dry mass per unit area of cotyledons sampled at the shaded site

### Data analyses

The power-law function was used to describe the scaling relationship between any two interdependent variables *Y* (e.g. leaf dry mass, *M*) and *X* (e.g. leaf area, *A*):


$$\begin{array}{l} Y=\beta X^{\alpha }, \end{array}$$ (1)

where β is the normalization constant, α is the scaling exponent (i.e. the slope of the log-log bivariate plot; Niklas 1994). If α = 1, two variables have a one-to-one (isometric) proportional relationship. If α ≠ 1, the scaling relationship is allometric. The log-transformation of the two sides of Equation (1) was used to stabilize the variance, i.e.


$$\begin{array}{l} \log Y=\gamma +\alpha \;\log X, \end{array}$$ (2)

where γ = log β (Niklas 1994). Reduced major axis regression protocols were used to estimate the regression parameters γ and α (Niklas 1994; Quinn and Keough 2002). The bootstrap percentile method (Efron and Tibshirani 1993; Sandhu *et al*. 2011) was used to obtain 3000 replicates of the scaling exponent α and calculate the 95% confidence intervals (CI) of α. This method was also used to test the significance of the difference between the scaling exponent of *M*_OPL_ versus *A*_OPL_ and the scaling exponent of *M*_SPL_ versus *A*_SPL_, as well as the difference between the scaling exponent of *M*_OC_ versus *A*_OC_ and the scaling exponent of *M*_SC_ versus *A*_SC_ (Table 1 for definition of symbols). Specifically, this method involves constructing bootstrap replicates for any two slope datasets and then subtracting them to determine if the 95 % CI of the difference includes 0. If the 95 % CI includes 0, it indicates no significant difference; otherwise, a significant difference exists between the two scaling exponents. *t*-tests were used to determine whether the trait values differed among the two light environments, i.e. *A*_OPL_ versus *A*_SPL_, *A*_OC_ versus *A*_SC_, *M*_OPL_ versus *M*_SPL_, *M*_OC_ versus *M*_SC_, LMA_OPL_ versus LMA_SPL_, LMA_OC_ versus LMA_SC_, *W*_OPL_/*L*_OPL_ versus *W*_SPL_/*L*_SPL_, and *W*_OC_/*L*_OC_ versus *W*_SC_/*L*_SC_ (Table 1 for definition of acronyms), and the differences were considered significant at *P* < 0.05. All statistical analyses were performed with R (version 4.3.2; R Core Team 2023).

## Results

### Allometry of primary leaves and cotyledons

**Figure 2.**
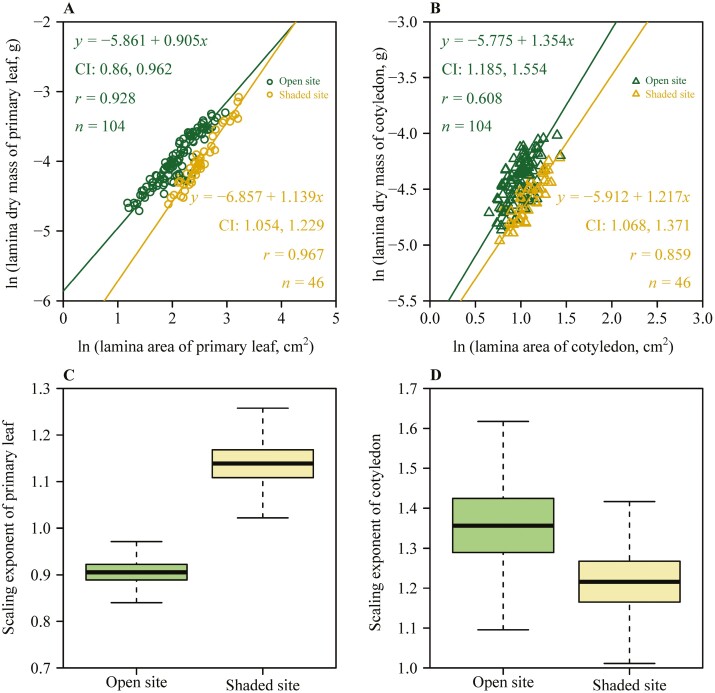
Linear fits of lamina dry mass of primary leaf versus lamina area of primary leaf (A), lamina dry mass of cotyledon versus lamina area of cotyledon (B), and boxplots of scaling exponent of primary leaf sampled at the open and the shaded sites (C) and boxplots of scaling exponent of cotyledon sampled at the open and the shaded sites (D) of *A*. *platanoides*. In panel (A) and (B), the data for both the *x*-axis and *y*-axis were logarithmically transformed, CI represents the 95% confidence intervals of the slope, *r* is the coefficient of correlation and *n* represents the number of leaves sampled. In panel (A), the green opened circles represent the primary leaves sampled at the open site, the yellow open circles represent the primary leaves sampled at the shaded site. In panel (B), the green open triangles represent the cotyledons sampled at the open site, the yellow open triangles represent the cotyledons sampled at the shaded site. In the boxplots, the horizontal solid line in each box represents the median; the whiskers extend to the most extreme data point, which is no more than 1.5 times the interquartile range from the box.

All the 95 % CIs of the scaling exponents of *M*_OPL_ versus *A*_OPL_, *M*_SPL_ versus *A*_SPL_, *M*_OC_ versus *A*_OC_, and *M*_SC_ versus *A*_SC_ did not include unity, indicating significant allometric scaling for *M*_OPL_ versus *A*_OPL_, *M*_SPL_ versus *A*_SPL_, *M*_OC_ versus *A*_OC_, and *M*_SC_ versus *A*_SC_ (Fig. 2A and B). The upper limit of the 95 % CI of the scaling exponents of *M*_OPL_ versus *A*_OPL_ did not exceed unity, indicating an ‘increasing returns’ phenomenon, i.e. a certain increase in mass resulted in a greater increase in lamina area. However, the lower bounds of the 95 % CIs of all the scaling exponents of *M*_SPL_ versus *A*_SPL_, *M*_OC_ versus *A*_OC_, and *M*_SC_ versus *A*_SC_ were greater than unity, i.e. increases in mass failed to keep pace with increases in area, indicating ‘diminishing returns’. Furthermore, the goodness of fit of the *M* versus *A* relationship for primary leaves was greater than that for the cotyledons (*r* = 0.928 for *M*_OPL_ vs. *A*_OPL_ compared by *r* = 0.608 for *M*_OC_ vs. *A*_OC_; and *r* = 0.967 for *M*_SPL_ vs. *A*_SPL_ compared by *r* = 0.859 for *M*_SC_ vs. *A*_SC_; Fig. 2A and B for details). The 95 % CI (i.e. −0.339, −0.130) of the differences between the scaling exponents of *M*_OPL_ versus *A*_OPL_ and *M*_SPL_ versus *A*_SPL_ did not include 0, indicating that there was a significant difference between the two scaling exponents. However, the 95% CI (i.e. –0.105, 0.390) of the differences between the scaling exponents of *M*_OC_ versus *A*_OC_ and *M*_SC_ versus *A*_SC_ included 0, which indicated that there was no significant difference between the two scaling exponents.

### Impacts of light availability on leaf traits

**Figure 3.**
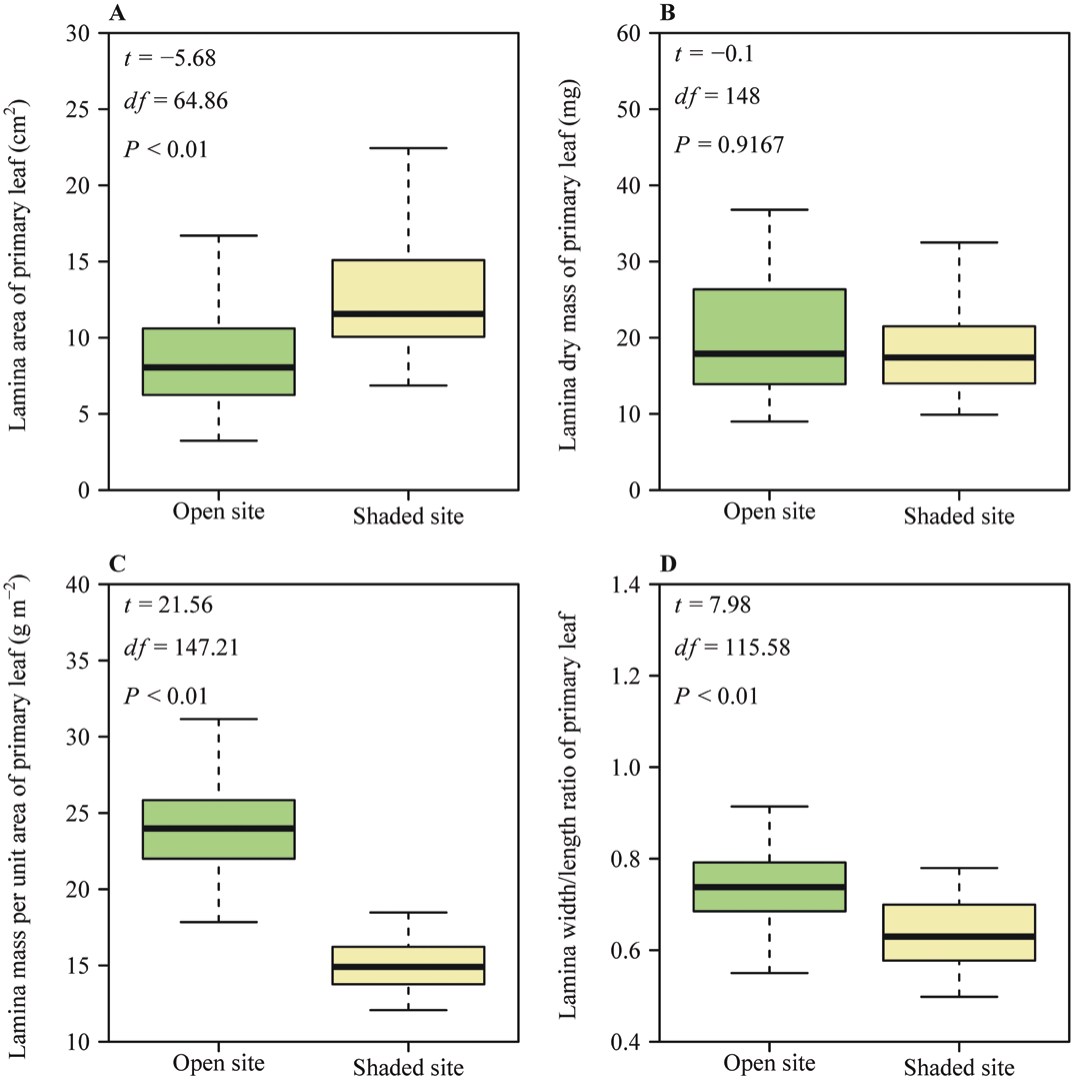
Boxplots of the lamina area of primary leaf (A), the lamina dry mass of primary leaf (B), lamina dry mass per unit area of primary leaf (C) and the lamina width/length ratio of primary leaf (D) in *A. platanoides* seedlings sampled from an open and a shaded site. Boxplot presentation as in Fig. 2. Trait averages among the open and shaded site were compared by *t*-tests.

For the primary leaves, the mean lamina area at the shaded site was greater than the mean lamina area at the open site (Fig. 3A). The lamina dry mass at the open site ranged from 9 to 36.8 mg, while the lamina dry mass at the shaded site ranged from 9.9 to 46 mg, and there were no significant differences between the two sites (Fig. 3B). The mean lamina mass per unit area at the open site was greater than that at the shaded site (Fig. 3C). The mean ratio of lamina width to length at the open site was greater than that at the shaded site (Fig. 3D).

**Figure 4.**
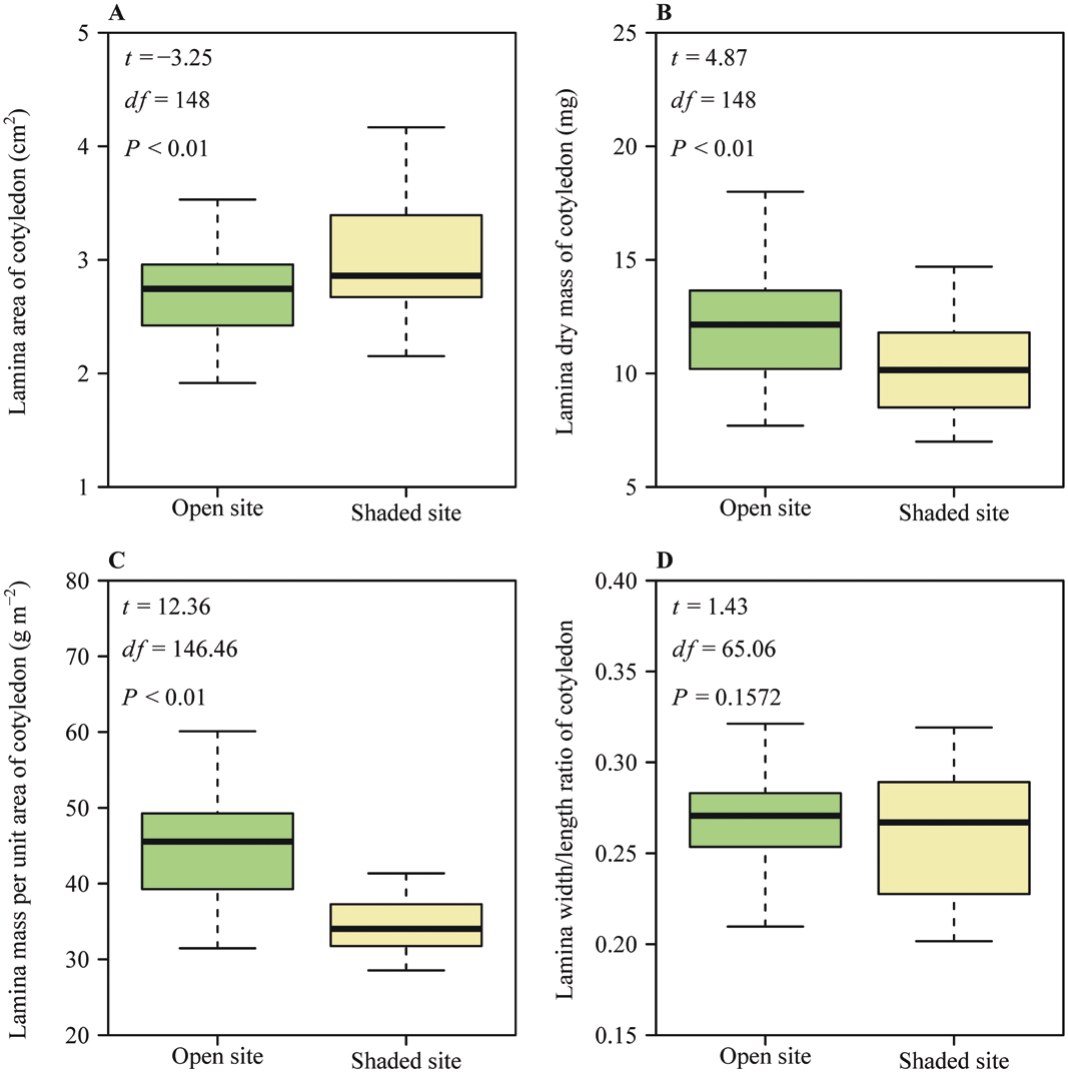
Boxplots of the lamina area of cotyledon (A), the lamina dry mass of cotyledon (B), the lamina dry mass per unit area of cotyledon (C) and the lamina width/length ratio of cotyledon (D) in *A. platanoides* seedlings sampled from an open and a shade site. Boxplot presentation as in Fig. 2 and statistical analysis as in Fig. 3.

For the cotyledons, the mean lamina area at the shaded site was greater than that at the open site (Fig. 4A). In contrast, the mean lamina mass at the open site was greater than that at the shaded site (Fig. 4B). The mean value of lamina mass per unit area at the open site was greater than that at the shaded site (Fig. 4C). The values of the ratio of lamina width to length at the open site ranged from 0.21 to 0.33, while the values of the ratio of lamina width to length at the shaded site ranged from 0.20 to 0.32, with no significant differences between the two sites (Fig. 4D).

## Discussion

### Scaling relationships of lamina dry mass versus lamina area of primary leaves and cotyledons

The data highlighted significant allometric relationships between lamina dry mass and lamina area both for primary leaves and cotyledons, i.e. the 95 % CIs of the scaling exponents of *M*_OPL_ versus *A*_OPL_, *M*_SPL_ versus *A*_SPL_, *M*_OC_ versus *A*_OC_, and *M*_SC_ versus *A*_SC_ did not include unity. The scaling exponents of *M*_SPL_ versus *A*_SPL_, *M*_OC_ versus *A*_OC_, and *M*_SC_ versus *A*_SC_ were larger than unity (Fig. 2A and B), indicating the phenomenon of ‘diminishing returns’ between lamina mass and lamina area; that is, increases in lamina area failed to keep pace with increases in lamina mass. However, the data for primary leaves from the open site, *M*_OPL_ versus *A*_OPL_, suggest that there is an ‘increasing returns’ relationship, i.e., increases in *A*_OPL_ exceeded increases in *M*_OPL_ (Fig. 2A).

Previous studies of the scaling relationship between leaf mass and area in mature trees have usually shown ‘diminishing returns’ (Niklas *et al*. 2007; Yang *et al*. 2020; Guo *et al*. 2021, 2023; Li *et al*. 2022), but we found ‘increasing returns’ between mass and area in the primary leaves of *A. platanoides* seedlings at high-light level (Fig. 2A). Compared to seedlings, mature trees have greater resource demands, and their resource allocation is more complex, requiring trade-offs between leaves, branches, roots and reproductive organs (Ogden 1974; Niklas and Enquist 2002; Liu and Ma 2015). Seedlings, being at an early stage of life, tend to optimize leaf structure to promote photosynthesis. After cotyledon senescence, the primary leaf takes over as the main sources for carbohydrates for plant growth (Zheng *et al*. 2011*a*). Generally, thicker leaves are associated with more strongly developed palisade cells, leading to enhanced photosynthetic capacity (Mendes *et al*. 2001; Terashima *et al*. 2001; Cai and Song 2005; Niinemets 2007*a*; Poorter *et al*. 2019; Niinemets 2020, 2023). Thus, our interpretation of the differences in scaling *M* versus *A* relationships depends on how lamina thickness varies within light environments with *A*. Although biomass input to seedlings grown at the open site leads to an exponential increase in primary lamina area, if relative thickness decreases with *A*, bigger leaves of given mass have potentially lower physiological activity per unit leaf area. By the same token, the opposite would be true for the shaded site. However, this reasoning does not consider the costs for within-leaf support. From the LMA (Leaf mass per area) data for the primary leaves (Fig. 3C), we conclude that the laminas of primary leaves are smaller and thicker at higher light levels and larger and thinner at lower light level (Niinemets *et al*. 2015; see below). Leaves with greater LMA are stiffer and thus, the costs for within-leaf support (fraction of structural tissue, fraction of epidermis) might be lower than for leaves with lower LMA (Niinemets 1999; Niinemets *et al.* 2007*b*). This reasoning suggests that in higher LMA leaves in higher light, greater leaf structural rigidity might allow a positive allometric scaling of *M* versus *A* without reduction of leaf physiological activity. Testing this hypothesis requires further study.

We observed that the ‘diminishing returns’ relationship was valid for cotyledons of *A. platanoides* at both shaded and open sites (Fig. 2B). At the initial stage of development, the cotyledons play a dual role, supplying of stored nutrients and carbohydrates to the developing seedling and capture of light and fixation of CO_2_ for further production of carbohydrates until the primary leaves are fully developed (Zheng *et al*. 2011*a*). The ‘diminishing returns’ relationship indicates that cotyledons with a greater mass have disproportionate lower area, and as such a potentially more superior storage function and inferior light interception function than cotyledons with a lower mass.

A developmental shift in the balance between the storage and supply functions of cotyledons can also partly explain the ‘diminishing returns’ observed for cotyledons in both light environments. At the time of sampling, the seedlings that we collected had almost fully expanded primary leaves. At this growth stage, the primary leaves were starting to serve as the main organ for photosynthesis of the seedling (Zheng *et al*. 2011*b*). Nevertheless, we cannot rule out that the smaller cotyledons were still expanding as the result of ongoing photosynthesis, whereas the bigger cotyledons were losing mass as the result of accelerating absorption of nutrients and carbon, explaining the ‘diminishing returns’ in both light environments.

### Effect of light availability on lamina traits of primary leaves and cotyledons

Our studied sites were in close vicinity to each other, but typically environmental factors vary along gap-understory spectrum, including variations in air humidity and temperature and soil nutrient and water availability (Bazzaz and Wayne 1994; Niinemets and Valladares 2004), and the question is to what extent the observed site differences in allometric scaling and leaf traits can be attributed to variation in light, and to what extent other environmental factors interfered with the effects of light. Despite the potential interaction of multiple environmental factors in our study, meta-analyses have demonstrated that light is the environmental factor most strongly affecting leaf structural characteristics, and the impact of other environmental drivers, e.g. nutrient availability is much less (Poorter *et al.* 2009; Niinemets *et al.* 2015). In fact, previous studies with *A. platanoides* along natural nutrient and light availability gradients (Niinemets 1998*a*) and in shade houses with different nutrient availability (Portsmuth and Niinemets 2007) demonstrate that light has the strongest control on leaf structural characteristics including the traits studied here. Furthermore, as the seedlings studied in our paper strongly rely on nutrient reserves in seeds, and the roots had not yet fully penetrated the turf layer in both sites, we argue that the probable soil differences were largely evened out at this stage of plant growth. Thus, we conclude that the site differences in leaf traits among the sites primarily reflect differences in light environment among the sites.

Interspecific variation in plant traits characterizes the adaptive responses of plants to their environment and plant survival strategies, whereas trait plasticity indicates plant responses to environmental variation (Ahrens *et al*. 2019; Thomas 2022; Wang *et al*. 2023). Among the four lamina traits of the primary leaf, dry mass showed no significant difference between the two light environments (Fig. 3B). However, the remaining three traits (i.e. *A*, LMA and *W*/*L*) exhibited significant differences between the two light environments (Fig. 3A, C, and D). In particular, the lamina area of primary leaf was smaller at high-light level (Fig. 3A). In combination with an approximate dry mass, this led to a greater LMA at higher light level (Fig. 3C), likely indicating that the leaves were thicker with greater photosynthetic capacity per unit area, in agreement with previous studies (Niinemets and Tenhunen 1997; Niinemets *et al*. 1998*b*, *c*; Niinemets *et al*. 2015; Feng *et al*. 2018; Choi 2021; Mohd Yusof *et al*. 2021). This strategy of reducing lamina area and increasing lamina thickness reduces transpiratory surface to maximize water use efficiency, simultaneously maximizing photosynthesis rate per area by having higher packing of photosynthetic cells per unit leaf area (Salisbury 1928; Parkhurst and Loucks 1972; Franks and Farquhar 2007; Okajima *et al*. 2012). In contrast, at low-light level, seedlings need to increase their lamina area to enhance the capture of light energy per unit biomass invested in leaves (Cheng *et al*. 2019). As a result, seedlings showed smaller LMA in low light consistent with previous studies (Liu and Liang 2016; Niinemets 2023). Considering that the leaf area was larger in low-light environment, the plant needs to invest more dry mass in support due to greater lever arms of more extended structures (Milla and Reich 2007; Niinemets *et al*. 2007*b*), explaining why the lamina dry mass of the primary leaf was comparable under both light conditions.

The ratio of leaf width to leaf length is commonly used as a leaf shape indice (Shi *et al*. 2015; Lin *et al*. 2020). The results indicate that the numerical value of the scaling exponent of *M* versus *A* decreases with an increasing quotient of leaf width and length of primary leaf. This is in accordance with the report of Lin *et al*. (2020), which showed a decreasing scaling exponent of leaf dry mass versus area with increasing lamina width/length ratio in 101 bamboo taxa. Furthermore, our study showed that the primary leaf became relatively wider at high-light level and relatively more extended at low-light level. When measuring the lamina length of the primary leaf, we defined it as the distance from the point of petiole attachment to the tip of the lamina, i.e. approximately equal to the lamina midrib length (Sack *et al*. 2013*a*). The leaf venation serves as the backbone of the lamina, and lamina area can increase only when the midrib length increases (Niinemets *et al*. 2007*b*; Sack *et al*. 2013*b*; Xiong *et al*. 2022). Given the long petioles in primary leaves of seedlings (Fig. 1B), having a greater leaf extension allows to reduce self-shading by allocating a greater fraction of leaf area further from stem axis (Pearcy and Yang 1998), but it is inevitably associated with greater support requirements due to greater lever arms. Thus, light-dependent differences in the shape of primary leaves as characterized by width/length ratio provide a further explanation of different scaling relationships (increasing vs. diminishing returns) at different light levels.

Out of four lamina traits of cotyledon, three traits (i.e. *A*, *M* and LMA) were significantly different between the two light environments (Fig. 4). Studies have demonstrated that cotyledons are capable of performing photosynthesis during the growth and development of seedlings (Brown and Huber 1987; Zhang *et al*. 2008; Ojeda *et al*. 2017; Shi *et al*. 2020). Our data suggested that the cotyledon dry mass was slightly lower at low light than at high light (Fig. 4B). This suggests that the light availability significantly limited cotyledon differentiation in our study. Despite a smaller mass, due to overall limited light availability, cotyledons contribute relatively more to overall plant growth and development under low-light conditions as reported by Jones (1959).

We observed minimal variability in the shape of cotyledons under the two light conditions (Fig. 4D), indicating that the shape of cotyledons was less strongly controlled by environmental drivers than the shape of primary leaves as has been observed in previous studies (Tang *et al*. 2022). This might indicate that cotyledon shape is already predetermined during the seed developmental period, implying a low phenotypic plasticity of this trait (Chandler 2008).

## Conclusions

Based on measurements of 104 primary leaves and 104 cotyledons from 52 seedlings of *A*. *platanoides* sampled from an open site and 46 primary leaves and 46 cotyledons from 23 seedlings sampled from a shaded site, indicated that the scaling relationships between lamina dry mass and lamina area had scaling exponents that significantly differed from unity. Statistically significant difference in the scaling exponents of *M*_OPL_ versus *A*_OPL_ and *M*_SPL_ versus *A*_SPL_ indicates a strong impact of light on the scaling relationship between lamina mass and area of the primary leaves of *A. platanoides* seedlings. Differently from previous studies, there was evidence that the primary leaves showed an ‘increasing returns’ phenomenon at the high-light level. ‘Diminishing returns’ relationships were found for both primary leaves and cotyledons sampled at the shaded site, as well as in cotyledons sampled at the open site. The data indicate that there are two different scaling relationships between lamina dry mass and lamina area of primary leaves and cotyledons of *A. platanoides* seedlings at the two light levels. At higher light, the lamina area was lower and LMA greater for both primary leaves and cotyledons. Additionally, the *W*/*L* of primary leaves was more susceptible to light, whereas the mass of cotyledons was more influenced by light. These findings provide novel insight into functional differences in primary leaves and cotyledons of seedlings growing in different forest light environments.

## Supporting Information

The following additional information is available in the online version of this article –

**Table S1.** The raw data of lamina dry mass, lamina area, lamina length, lamina width of *Acer platanoides* L. seedlings.

plae054_suppl_Supplementary_Tables

## Data Availability

All data supporting the finding of this study are available as supporting information.
